# Subjective memory complaints among patients on sick leave are associated with symptoms of fatigue and anxiety

**DOI:** 10.3389/fpsyg.2015.01338

**Published:** 2015-09-08

**Authors:** Julie K. Aasvik, Astrid Woodhouse, Henrik B. Jacobsen, Petter C. Borchgrevink, Tore C. Stiles, Nils I. Landrø

**Affiliations:** ^1^Department of Circulation and Medical Imaging, Faculty of Medicine, Norwegian University of Science and TechnologyTrondheim, Norway; ^2^Hysnes Rehabilitation Center, St. Olav’s University HospitalTrondheim, Norway; ^3^National Competence Centre for Complex Disorders, St. Olav’s University HospitalTrondheim, Norway; ^4^Department of Public Health and General Practice, Norwegian University of Science of TechnologyTrondheim, Norway; ^5^Department of Psychology, Norwegian University of Science and TechnologyTrondheim, Norway; ^6^Clinical Neuroscience Group, Department of Psychology, University of OsloOslo, Norway

**Keywords:** cognitive, complaints, concurrent, symptoms, sick leave

## Abstract

**Objective:** The aim of this study was to identify symptoms associated with subjective memory complaints (SMCs) among subjects who are currently on sick leave due to symptoms of chronic pain, fatigue, depression, anxiety, and insomnia.

**Methods:** This was a cross-sectional study, subjects (*n* = 167) who were currently on sick leave were asked to complete an extensive survey consisting of the following: items addressing their sociodemographics, one item from the SF-8 health survey measuring pain, Chalder Fatigue Questionnaire, Hospital Anxiety and Depression Scale, Insomnia Severity Index, and Everyday Memory Questionnaire – Revised. General linear modeling was used to analyze variables associated with SMCs.

**Results:** Symptoms of fatigue (*p*-value < 0.001) and anxiety (*p*-value = 0.001) were uniquely and significantly associated with perceived memory failures. The associations with symptoms of pain, depression, and insomnia were not statistically significant.

**Conclusions:** Subjective memory complaints should be recognized as part of the complex symptomatology among patients who report multiple symptoms, especially in cases of fatigue and anxiety. Self-report questionnaires measuring perceived memory failures may be a quick and easy way to incorporate and extend this knowledge into clinical practice.

## Introduction

Subjective memory complaints (SMCs) have been associated with conditions of chronic pain (McCracken and Iverson, 2001; Katz et al., 2004; Munoz and Esteve, 2005; Tesio et al., 2014), fatigue (Cope et al., 1995; Suhr, 2003; Roth et al., 2005), depression (Comijs et al., 2002; Roth et al., 2005; Mowla et al., 2007), and anxiety (O‘Boyle et al., 1990; Derouesne et al., 1999) as well as insomnia (Kronholm et al., 2009; Magali et al., 2012). The fact that all of the aforementioned conditions also often co-occur (Smith, 1992; Schotte et al., 2006; Stewart et al., 2006; Friedberg, 2010; Lumley et al., 2011) adds to the complexity of studying the nature of SMCs. SMCs include forgetfulness, increased distractibility and reduced mental alertness. Self–perceived memory problems are widespread in the general population, and estimates show that they are prevalent in all age groups and in both genders (Cooper et al., 2011; Holmen et al., 2013). The estimates increase when SMCs are associated with illness (Cockshell and Mathias, 2014).

Subjective memory complaints are considered as one of the major diagnostic criteria’s for depression (DSM-5). It has been associated with mild cognitive impairment (Erk et al., 2011) and has been highlighted as a potential marker for future dementia and Alzheimer’s disease (Stewart et al., 2011; Stewart, 2012). SMCs have also been associated with work-related exhaustion (Ekstedt and Fagerberg, 2005) and impaired ability to cope with work-related stress (Broadbent et al., 1982). Despite the frequency of SMCs and their potential negative impacts, SMCs remain one of the least recognized and assessed aspects within the field of occupational health psychology.

A number of studies have reported associations between SMCs and depression (McCracken and Iverson, 2001; Comijs et al., 2002; Roth et al., 2005; Mowla et al., 2007). It has been suggested that this relationship might reflect an extension of the emotional distress, low self-esteem, and lack of confidence associated with depression. Such factors might cause tendencies to underestimate one’s own performance and, consequently over-report memory failures. Supporting this view, Antikainen et al. (2001) found that mood improvement was associated with reduced SMCs in depressed individuals. Landrø et al. (2013) found higher rates of SMCs in patients with chronic pain, even after adjusting for symptoms of depression. This indicates that the interpretation of SMCs as solely an expression of depressive symptomatology might be somewhat incomplete. In addition, one study (Derouesne et al., 1999) found that the best predictor of the severity of SMCs included symptoms of anxiety. However, although a number of studies have included measures of depression, few have considered co-morbid anxiety.

Studies of SMCs in patients with chronic pain have reported inconsistent results for the associations with pain intensity (Comijs et al., 2002; Munoz and Esteve, 2005; Tesio et al., 2014). Moreover, some studies have found a correspondence between SMCs and pain-related anxiety and pain catastrophizing (McCracken and Iverson, 2001; Munoz and Esteve, 2005), suggesting that it is not just pain intensity *per se* that is associated with SMCs. Studies examining the associations between chronic pain and SMCs also differ in regards to whether they have included measures of fatigue even though these symptoms often co-occur. Studies on the association between SMCs and insomnia have yielded somewhat contradicting results (Kronholm et al., 2009; Magali et al., 2012).

In general, the multi-factorial nature of SMCs is challenging, and what seems to be lacking is a more comprehensive examination of all relevant symptom dimensions that are associated with SMCs. Identifying key symptoms associated with SMCs might have clinical implications by aiding therapeutic and rehabilitation programs to develop interventions that target the specific conditions that are associated with such complaints. In this study, the symptoms of pain, fatigue, depression, anxiety, and insomnia as well as self-reported memory complaints were assessed in a sample of patients on sick leave who were enrolled in a vocational rehabilitation program. The main aim of the study was to identify which of the five symptoms is significantly associated with subjective memory complaint.

## Materials and Methods

### Ethics Statement

All subjects received information about the study and gave written informed consent before inclusion. The study was approved by Regional Committees for Medical and Health Research Ethics – REC Central, and it was conducted in accordance with the declaration of Helsinki.

### Study Design and Setting

This was a cross-sectional study, conducted during the period of January 2012 until March 2013. Participants were consecutively recruited from an inpatient vocational rehabilitation center in Norway.

General practitioners referred patients who were on sick leave to a 3.5-weeks intervention at a vocational rehabilitation center. All patients were asked to answer a web-based survey before meeting with a multidisciplinary team (physician, psychologist, and physiotherapist) at a designated outpatient clinic to determine if they met the inclusion criteria. The survey included measures of socio-demographics, pain, fatigue, depression, anxiety, sleep disturbances and SMCs. The collected data were used as a source tool by the outpatient and inpatient clinics and were simultaneously included in a research database.

### Study Participants

All participants were on sick leave (<8 weeks) due to chronic pain, fatigue and/or common mental disorders. All participants were between 18–59 years of age. The exclusion criteria were patients with severe mental disorders, (acute psychosis, ongoing manic episode, or suicidal ideation) ongoing substance abuse or addiction, cases of pregnancy, or individuals who were unable to communicate in Norwegian. Considering that this was a return-to-work rehabilitation evaluation, patients were excluded if they did not define returning to work as a personal goal. For further details on the intervention, see (Fimland et al., 2014).

### Assessments

#### Memory Complaints

Subjective cognitive complaints are based upon a self-report questionnaire, The Everyday Memory Questionnaire-Revised (EMQ-R; Royle and Lincoln, 2008). The EMQ was originally developed by Sunderland et al. (1983) and later revised (shortened) by Royle and Lincoln (2008). The EMQ-R consists of 13 items, it makes it possible to distinguish between three factors, two of them have been identified as (1) retrieval and (2) attentional tracking, the third has not been identified or labeled. Each question is rated on a 5-point scale ranging from A, scored as zero – “Once or less in the last month,” to E, scored as four – “Once or more in a day.” The items were summed, giving a scale from 0 to 52. Because we chose to focus on general memory functioning we used the total sum score divided by the number of items, following the scoring procedure. Reliability tests on the EMQ have shown a strong internal reliability, with a Cronbach’s alpha score of 0.89 (Royle and Lincoln, 2008).

#### Chronic Pain

We used one item from the SF- 8 health survey for measuring the intensity of pain (Ware et al., 2001). The subjects were asked “How much bodily pain have you experienced during the last week?” (none, very mild, mild, moderate, severe, or very severe). As this is a single item measure, alpha values are not applicable. The item has shown an intra-class correlation coefficient of 0.66 (95% CI 0.65–0.67; Landmark et al., 2012).

#### Depression and Anxiety

The Hospital Anxiety and Depression Scale (HADS) was used (Zigmond and Snaith, 1983). The fourteen item scale is divided into two sub-scales; one sub-scale scores depression and the other scores anxiety. Each item ranges from 0 to 3, and the items are summed, giving a score ranging from 0 to 21 in each sub-scale. The psychometric properties of the scale as a self-rating instrument have been validated (Olssøn et al., 2005). In a review of HADS the correlations between the two subscales varied from 0.40 to 0.74 (mean 0.56). Cronbach’s alpha for HADS-A varied from 0.68 to 0.93 (mean 0.83) and for HADS-D from 0.67 to 0.90 (mean 0.82; Bjelland et al., 2001).

#### Fatigue

Fatigue was measured with The Chalder Fatigue Questionnaire (CFQ Chalder et al., 1993), which consists of 11 questions, reflecting physical, and mental fatigue. Each item has four response categories that are scored bimodally 0-0-1-1. Responses are summed in a scale from 0 to 11. The scale is both reliable and valid and has been validated for a Norwegian population. Cronbach’s alpha has been calculated for all items (range 0.88–0.90). Split half reliability has also been calculated (0.8613 and 0.8466, respectively) in a previous study (Chalder et al., 1993; Loge et al., 1998; Moriss et al., 1998).

#### Insomnia

The Insomnia Severity Index (ISI) was used (Bastien et al., 2001). This is a self-rated questionnaire consisting of seven items designed to assess insomnia severity by the following: difficulty falling asleep, night time awakenings, early morning awakenings, impairment of daytime functioning due to sleep disturbances, noticeability of problems, distress, or worry caused by sleep disturbances, and dissatisfaction with sleep. Each item is rated according to a 5-point scale, ranging from 0 (not at all) to 4 (very much). The items were summed, giving a scale of 0–28. The ISI is a reliable, valid and sensitive self-report questionnaire, and has been recommended as a screening device and an outcome measure in insomnia treatment and insomnia research. The internal consistency of the ISI was reported to be 0.74 (Bastien et al., 2001; Morin et al., 2011).

### Statistical Analyses

Data was analyzed using the Statistical Package for the Social Sciences (SPSS version 20.0; IBM Corporation, Armonk, NY, USA). To test the association between symptom measures we used Spearman‘s correlation. General linear modeling (GLM) was used to analyze variables that were associated with SMCs. However, the variables had unacceptable residual plots, and as a result multiple robust regression with Huber’s correction was chosen in both unadjusted and adjusted models. Each independent variable (fatigue, pain, anxiety, depression, and insomnia) was first entered in a separate analysis while controlling for age and gender. In *post hoc* analyses the variables that were significantly associated with SMCs were simultaneously entered while controlling for age and gender in order to assess the unique associations with SMCs.

## Results

### Participant Characteristics

The mean age for the entire group was 41 years (*SD* 9.9), and 78.3 % of the subjects were women. Of the 198 patients who were originally included, seven subjects declined to participate, and 8 subjects canceled their rehabilitation intervention and withdrew from the study due to illness or injury. Sixteen subjects had missing data on the Everyday Memory Questionnaire; they were excluded case by case from the analyses. Analyses confirmed that the excluded subjects were similar to the sample population in terms of their symptoms of chronic pain, fatigue, anxiety, depression, and insomnia. The final study population consisted of 167 subjects. The distribution of diagnoses in the sample was: chronic pain 36.8% (*n* = 63), fatigue 28.7% (*n* = 49), depression 11.1% (*n* = 19), anxiety 4.7% (*n* = 8), both depression and anxiety 4.7% (*n* = 8), other diagnoses (including obesity, diabetes, hypothyroidism, asthma etc.) 15.2% (*n* = 26). None of the subjects had a diagnosis of insomnia or sleep-disturbances. 25.7% (*n* = 44) were referred based on a description of their health complaints rather than a specific diagnosis. 23.4% (*n* = 40) of the sample had two diagnoses, 4.7% (*n* = 8) had three diagnoses, underlining the fact that this group of subjects often present multiple, non-specific and complex health-related complaints.

Self-reported complaints regarding everyday memory failures, chronic pain, fatigue, depression, anxiety, and insomnia are described in **Table 1**. The average score on EMQ-R indicate that our subjects experience multiple kinds of memory failures several times a month, but not on a weekly basis according to the prevailing interpretation of the questionnaire (Royle and Lincoln, 2008). The mean score on the item assessing pain intensity (SF8) indicate (according to the scale) that subjects have experienced mild to moderate pain in the last 4 weeks. The average score on CFQ is above the cut-off at ≥5 used to indicate clinical fatigue (Loge et al., 1998). Subjects score slightly higher on measures of anxiety than depression. Average scores are just below the cut-off at ≥8 used to indicate clinical anxiety and depression (Haug et al., 2004). Finally, the average score on ISI is within the range (8–14) of what constitutes as subthreshold insomnia (Bastien et al., 2001).

**Table 1:** Means and *SD* of the various symptom measures.

Self-Report Questionnaires	*N*	*M*	*SD*
Everyday Memory Complaints (EMQ-R)	167	1.3	0.9
Chronic pain (SF8)	167	3.6	1.2
Chalder Fatigue Scale (CFS)	164	8.1	2.7
The Hospital Anxiety and Depression Scale (HADS)			
HADS Anxiety	167	7.5	4.1
HADS Depression	167	5.6	3.8
Insomnia Severity Index (ISI)	167	10.6	5.9

Because of the comorbidity when it comes to symptoms of chronic pain, fatigue, depression, anxiety, and insomnia we calculated the inter-correlations between the symptom measures, the results are described in **Table 2**. We found a significant correlation between fatigue, depression, anxiety, and insomnia, while pain intensity was significantly correlated with insomnia.

**Table 2:** Inter-correlations between the symptom measures.

	1	2	3	4	5
(1) Insomnia	-	-	-	-	-
(2) Pain	0.334^∗∗^	-	-	-	-
(3) Fatigue	0.248^∗∗^	0.146	-	-	-
(4) Depression	0.266^∗∗^	0.052	0.276^∗∗^	-	-
(5) Anxiety	0.268^∗∗^	0.088	0.177^∗^	0.569^∗∗^	-

*^∗^Correlation is significant at the 0.05 level 2-tailed, ^∗∗^Correlation is significant at the 0.01 level 2-tailed*.

As shown in **Table 3**, the initial unadjusted analyses showed that both levels of fatigue, anxiety, and depression were significantly associated with SMCs. When these three variables were entered simultaneously with age and gender as covariates in *post hoc* analyses the levels of fatigue and anxiety remained significantly associated with SMCs but levels of depression were no longer significantly associated with SMCs. Adjusted *R*^2^ showed an explained variance of 22%.

**Table 3:** A summary of the statistical results.

Variables	β-estimate (Adjusted age and gender)	95% CI	*P*-value	*P*-estimate (Adjusted sign variables)	95% CI	*P*-value
Age	0.002	(-0.012-0.016)	0.321	0.003	(-0.010-0.017)	-
Men vs. Women	0.058	(-0.247-0.364)	0.473	0.089	(-0.022-0.399)	-
Fatigue + 1 on CFS	0.150	(0.095-0.206)	**<0.001**	0.153	(0.081-0.190)	**<0.001**
Anxiety + 1 on HADS	0.073	(0.043-0.104)	**<0.001**	0.062	(0.027-0.097)	**0.001**
Depression + 1 on HADS	0.054	(0.018-0.091)	**0.004**	-0.009	(-0.050-0.032)	0.524
Pain + 1 on SF8	0.027	(-0.288-0.342)	0.969	-	-	-
Insomnia + 1 on ISI	0.022	(-0.001-0.046)	0.067	-	-	-

*Adjusted *R*-squared = 0.226*.

*Robust multiple regression using Huber’s correction was chosen for fatigue and in the fully adjusted model*.

*Bold values indicate significant findings*.

## Discussion

Levels of fatigue and anxiety were significantly and uniquely associated with SMCs, while levels of depression, insomnia, and pain intensity were not. The strong inter-correlations between the independent variables underlines the importance of accounting for all known comorbidities when trying to identify the predictors of SMCs. Levels of depression were significantly associated with SMCs when entered separately into the analysis, but it did not remain significant when levels of fatigue and anxiety were entered into the equation. This suggests that depression is not uniquely associated with SMCs; it rather seems to be secondary to symptoms of fatigue and anxiety.

Subjective memory complaints might reflect worries about one’s own memory performance, making it an expression of anxiety. Previous studies have reported that memory related anxiety, experiencing anxious arousal in response to forgetting, becoming anxious when asked to remember something and worrying about one’s own memory function corresponds to SMCs (Ponds and Jolles, 1996; Jorm et al., 2001; Mol et al., 2008). It remains unclear whether SMCs originate from dispositional tendencies to worry or cause worry due to increased vigilance of one’s own cognitive performance, as our data do not provide any information about the cause and effect. SMCs may be the final product of the ongoing competition for attention. Anxiety consumes attention, which could be the underlying cause of SMCs because preoccupation with excessive thoughts of worry could disturb the processing of other information.

Our findings are in line with other studies that have reported a strong association between fatigue and SMCs (O‘Boyle et al., 1990; Cope et al., 1995; Derouesne et al., 1999; Williams et al., 2011). Chronic fatigue is characterized by the sensation of persistent and debilitating tiredness and exhaustion. While acute fatigue decreases and disappears after periods of rest, moderate to long-term fatigue does not respond to the same restorative techniques. Although the specific mechanisms underlying the association between fatigue and SMCs are unknown, one can speculate on whether the lower activation, sensations of tiredness, and exhaustion might explain this link. Fatigue might reduce the ability to mobilize and utilize effort to help regulate cognitive performance, contributing to the perception of cognitive decline.

Biased attention toward health-related threat stimuli and illness-related impairments among patients suffering from chronic fatigue has been reported (Ray et al., 1995; Aggrawal et al., 2006; Hou et al., 2008). The attentional bias toward signals of potential threat indicates some degree of anxiety, which is supported by the high comorbidity of fatigue and generalized anxiety disorder (Fischler et al., 1997). While effort is known to compensate for some of the cognitive difficulties associated with anxiety (Eysenck et al., 2007), symptoms of fatigue make it inherently difficult to mobilize and use the same compensational technique. The inability to tap into and utilize auxiliary processing resources might be the characteristic feature that differentiates fatigue from anxiety with respect to subjective memory functioning.

Both the distribution of diagnoses and measures on the self-report questionnaires show that subjects present multiple non-specific health-related complaints. Fatigue and chronic pain were the most common diagnoses followed by depression and anxiety. None of the subjects in the sample were referred based on a diagnosis of insomnia or sleep disturbances, still the mean score of the sample on ISI are within subthreshold insomnia. The mean score on the EMQ-R was higher than what has been found in MS- patients (Royle and Lincoln, 2008). This is somewhat surprising given the fact that the latter suffers from a neurodegenerative disease known to affect cognitive functioning (Bobholz and Rao, 2003) and brain morphology (Papadopoulos et al., 2009).

It has been reported that nurses in high stress wards with high scores on the Cognitive Failure Questionnaire had greater difficulties coping with stress and reported more emotional distress than nurses with low scores on the questionnaire (Broadbent et al., 1982). One study found that self-reported cognitive decline precedes work-stress-related exhaustion (Ekstedt and Fagerberg, 2005). High scores on self-reported cognitive dysfunction might indicate vulnerability in terms of experiencing work-related stress (Folkman et al., 1986). This line of research seems to support our notion that subjects experiencing fatigue might find it difficult to access and make use of cognitive resources in order to cope with or adjust to various demands of everyday life, this might in turn explain their perceptions of impaired memory functioning. If SMCs reflect a lack of accessibility to cognitive resources, it might also affect coping and induce or increase stress and thereby become a catalyst for sick leave.

Our sample reported multiple symptoms, the co-occurrence of symptoms adds to the total symptom burden and the added morbidity is associated with sick leave (Aamland et al., 2012). SMCs are part of the rather complex equation and should be addressed in clinical practice. Self-report questionnaires measuring perceived memory functioning is a quick and easy way to introduce and assess SMCs in clinical practice. For some patients who report significant problems with memory it might be relevant to do a neuropsychological examination in order to get objective measures and assess specific cognitive functions. Some patients might also benefit from cognitive training. Some studies have reported promising results when it comes to the effects of cognitive training (Klingberg, 2010), while others have not (Shipstead et al., 2012).

Future research should examine the relationship between SMCs stress and coping, as it might provide new insight into important mechanisms with respect to occupational functioning and sick leave.

### Limitations

The current study population is a small and selected sample, which limits the generalizability of the results. The results should be interpreted with caution as 16 of the originally eligible subjects had missing data on the EMQ and was excluded from the study. We used one item from SF8 Health Questionnaire to assess the level of pain. Considering that pain is a multidimensional experience we should have included more information in order to tap into the multifactorial nature of pain. The explained variance of the model was 22%, which means that 78% of the variance when it comes to SMCs is explained by other factors than the ones examined in this study.

## Conclusion

Subjective memory complaints should be included as part of the complex symptomology among patients who report multiple symptoms, especially in cases with symptoms of fatigue and anxiety. This knowledge should be incorporated into clinical practice and questionnaires assessing perceived memory failures may be an easy and effective way to get patients to communicate about their perceptions and experiences when it comes to this aspect of cognitive functioning and occupational health.

Our results also emphasize the importance of accounting for all known comorbidities when examining SMCs in patients with multiple symptoms.

## Conflict of Interest Statement

The authors declare that the research was conducted in the absence of any commercial or financial relationships that could be construed as a potential conflict of interest.
